# Myeloid Differentiation Primary Response 88–Cyclin D1 Signaling in Breast Cancer Cells Regulates Toll-Like Receptor 3-Mediated Cell Proliferation

**DOI:** 10.3389/fonc.2020.01780

**Published:** 2020-09-18

**Authors:** Aradhana Singh, Ranjitsinh Devkar, Anupam Basu

**Affiliations:** ^1^Molecular Biology and Human Genetics Laboratory, Department of Zoology, The University of Burdwan, Bardhaman, India; ^2^Department of Zoology, Faculty of Science, The M.S. University of Baroda, Vadodara, India

**Keywords:** TLR3, MyD88, cancer, breast cancer, Poly(I:C), IRAK1, NFkB, IL-6

## Abstract

Toll-like receptor 3 (TLR3)-mediated apoptotic changes in cancer cells are well-documented, and hence, several synthetic ligands of TLR3 are being used for adjuvant therapy, but there are reports showing a contradictory effect of TLR3 signaling, which include our previous report that had shown cell proliferation following surface localization of TLR 3. However, the underlying mechanism of cell surface localization of TLR3 and subsequent cell proliferation lacks clarity. This study addresses the TLR3 ligand-mediated signaling cascade that regulates a proliferative effect in breast cancer cells (MDA-MB-231 and T47D) challenged with TLR3 ligand in the presence of myeloid differentiation primary response 88 (MyD88) inhibitor. Evidences were obtained using immunoblotting, coimmunoprecipitation, confocal microscopy, immunocytochemistry, ELISA, and flow cytometry. Results had revealed that TLR3 ligand treatment significantly enhanced breast cancer cell proliferation marked by an upregulated expression of cyclinD1, but the same was suppressed by the addition of MyD88 inhibitor. Also, expression of interleukin 1 receptor-associated kinase 1 (IRAK1)–TNF receptor-associated factor 6 (TRAF6)–transforming growth factor beta-activated kinase 1 (TAK1) was altered in the given TLR3-signaling pathway. Inhibition of MyD88 disrupted the downstream adaptor complex and mediated signaling through the TLR3–MyD88–NF-κB (p65)–IL-6–cyclin D1 pathway. TLR3-mediated alternative signaling of the TLR3–MyD88–IRAK1–TRAF6–TAK1–TAB1–NF-κB axis leads to upregulation of IL6 and cyclin D1. This response is hypothesized to be via the MyD88 gateway that culminates in the proliferation of breast cancer cells. Overall, this study provides first comprehensive evidence on the involvement of canonical signaling of TLR3 using MyD88–cyclin D1-mediated breast cancer cell proliferation. The findings elucidated herein will provide valuable insights into understanding the TLR3-mediated adjuvant therapy in cancer.

## Introduction

Toll-like receptor 3 (TLR3) recognizes double-stranded RNA (dsRNA) of viral origin, small interfering RNAs, and self-RNAs derived from damaged cells (1, 2). TLR3 induces potent antigen-specific CD8+ T-cell responses that directly induce effector CD8+ T cell and natural killer (NK) cells for IFN-γ release (3). TLR3 was reported to be expressed not only by immune cells but also in the various cancer cells, such as breast cancer (4), prostate cancer (5), epithelial adenocarcinoma (6), and others. Classically TLR3 signaling is mediated through the endosomal compartment of the cells. Intracellular TLR3 signaling can directly induce apoptosis (3, 4, 7). The TLR3 structure comprises a leucine-rich repeat domain, a transmembrane region, a linker region, and a Toll/IL-1 receptor (TIR) domain (8, 9). Ligand binding is mediated by the leucine-rich domain, whereas intracellular signaling is propagated by the TIR domain (3, 4).

Canonical TLR signaling has been reported to be regulated by an array of molecules through various mechanisms to adjust the consequences of associated autoimmune and inflammatory diseases. In the canonical pathway, for most of the TLRs, upon ligand activation, MyD88 is recruited as a dimer in the cytoplasmic TIR domain in a homophilic interaction (10–15).

TLR3 agonists have been used in immunotherapy for various clinical and preclinical studies. The majority of clinical studies establish TLR3 as a tumor suppressor using synthetic ligand polyinosinic:polycytidylic acid [poly(I:C)] or poly-ICLC for adjuvant therapy or targeted therapy (16–19). Ligand binding has been reported to induce endosomal TLR3-mediated recruitment of TIR domain-containing adapter-inducing interferon β (TRIF) (20) to trigger type-I IFN and to induce cellular apoptosis (3–5, 21, 22). On the contrary, TLR3 has been reported to be highly expressed in breast tumors and is associated with poor prognosis of the disease (13, 23, 24).

Induction of cell proliferation via surface localization of TLR3 has been shown by our research group in breast cancer cells (25) and by other groups in diverse types of cancers (1, 26). This mechanism is supposedly an alternative to the endosomal-mediated action, but the exact mechanism of alternating TLR3 signaling lacks clarity. In this study, we have addressed the alternative signaling, independent of TRIF activation, to decipher the mechanistic cascade of alternative cellular proliferative mode of TLR3 signaling.

## Materials and Methods

### Cell Lines and Cell Culture Conditions

Human breast cancer cells MDA-MB-231 and T47D were obtained from the National Center for Cell Science, Pune, India. MDA-MB-231 cells were cultivated in L-15 medium (Himedia, India), and T47D cells were grown in RPMI 1640. All the media were supplemented with 10% FBS (GIBCO) and 1% L-glutamine–penicillin–streptomycin (200 mM L-glutamine, 10,000 U/ml of penicillin, and 10 mg/ml of streptomycin; Himedia, India). T47D cells were maintained at 37°C in a humidified incubator with 5% CO_2_, while MDA-MB-231 cells were maintained at 37°C in a humidified incubator without CO_2_.

### TLR3 Ligand

Poly(I:C) HMW (Catalog no. tlrl-pic; Invivogen) was used as a synthetic ligand of TLR3. Accordingly, a dose of 10 μg/ml of Poly(I:C) was used in serum-free culture media to bind with TLR3 present in the cell.

### MyD88 Inhibitor

MyD88 inhibitor ST2825 (MCE-HY-50937) was used to block the dimerization of MyD88. Cells were treated with ST2825 (1 μM), for 4 h prior to the addition of poly(I:C).

### Cell Viability Assay

Breast cancer cells were seeded in a 35-mm culture dish at a density of 40 × 10^4^ cells in appropriate culture media supplemented with 10% FBS and allowed to grow for 24 h. After the cells have reached nearly 40–50% confluence, cells were pretreated with MyD88 inhibitor for 4 h. Then the TLR3 ligand was added to the serum-free media and incubated for 24 h. At the end of the incubation period, cells were trypsinized and stained with trypan blue, and the number of viable cells was counted under a microscope. Two technical replicates per sample were run in each independent experiment.

### BrdU Incorporation Assay

To confirm active DNA synthesis as a confirmatory index of cellular proliferation, BrdU incorporation assay was carried out through flow cytometry. Briefly, cells were plated in a 35-mm culture dish at 40 × 10^4^ cells per dish and allowed to adhere overnight in complete media at 37°C. The cells were treated with MyD88 inhibitor for 4 h prior to the addition of the TLR3 ligand. Cells were incubated for 24 h. At the end of the culture, 10 μM BrdU (BD Pharmingen BrdU Flow Kit, San Diego, CA, USA) was added, and the target cells were incubated for another 30 min, the medium was discarded, and the cells were fixed at room temperature for 30 min. Cells were permeabilized, and FITC conjugate anti-BrdU antibody (BD Pharmingen) was allowed to bind with the incorporated BrdU. After washing, cells were incubated with 7AAD and acquired through BD FACSVerse flow cytometer (BD Biosciences, San Diego, CA, USA).

### Immunocytochemistry

To check the expression of TLR3 in the cell surface as well as the level of IL-6 in the cytoplasm, immunocytochemistry was performed. Briefly, 40 × 10^4^ cells were seeded on a coverslip in a 35-mm culture dish in complete media. Cells were allowed to adhere overnight and treated. Four hours before the addition of the TLR3 ligand, the MyD88 inhibitor was added and incubated for 24 h. For TLR3 surface expression, cells were fixed and allowed to bind with TLR 3 antibody (Invitrogen-PA5-29619) and Alexa 594-conjugated secondary anti-rabbit goat antibody (Invitrogen-A11012). For IL-6 expression, cells were fixed, permeabilized, and incubated with primary IL-6 antibody (Invitrogen-AMC0862) and Alexa 488-conjugated anti-mouse goat antibody (Invitrogen-A11001) and mounted with Vecta Shield-DAPI to counterstain the nuclei and were observed under fluorescence microscope (Leica DMI 6000B).

### Confocal Microscopy Study

Confocal microscopy was carried to study the change in nuclear localization of NF-κB in breast cancer cells after TLR3 ligand activation. Cells were seeded with a density of 40 × 10^4^ on a coverslip with a 35-mm culture plate. Cells were treated with TLR3 ligand with or without MyD88 inhibitor for 30, 60, and 90 min. Cells were fixed with 3% PFA (paraformaldehyde solution) for 15 min at room temperature, washed with PBS and transferred to 100% methanol for 5 min, and washed with PBS and permeabilized with PBS containing 0.25% Triton X-100 for 5 min. After fixation and permeabilization, blocking was done using PBS containing 1% BSA for 1 h. After blocking, cells were allowed to bind with the NF-kB p65 antibody (Invitrogen-PA1-186) overnight at 4°C followed by anti-rabbit antibody conjugated with Alexa-594 for 1 h at room temperature in the dark. Coverslips were mounted with Vecta Shield-DAPI to counterstain the nuclei and analyzed by Zeiss LSM 710 inverted confocal microscope (Zeiss, Germany) with a 63X plan apochromat objective. Image analysis was performed using the ImageJ v3.91 software (http://rsb.info.nih.gov/ij).

### ELISA

IL-6 was quantified from the cell supernatant of the challenged cells by ELISA. Briefly, cells were seeded at a density of 100 × 10^4^ cells in a 35-mm culture plate and grown to confluence. The confluent monolayer was washed twice and kept in media supplemented with 1% ITS. Then incubation with TLR3 ligand and MyD88 inhibitor was performed for 36 h. Two replicates per sample were run in each independent experiment. At the end of incubation, the condition media were collected, and the quantity of the secretory IL-6 was estimated using a commercially available ELISA kit (R&D Systems, DY 206-05) with human IL6 antibody (R&D Systems, DY 008).

### Western Blotting

Cells were cultured at a density of 100 × 10^4^ cells in a 60-mm culture plate (Tarsons-960020). Four hours before the addition of the TLR3 ligand, the MyD88 inhibitor was added and incubated for 24 h. Cells were lysed using RIPA buffer, and gel electrophoresis was performed using acrylamide gel. Proteins were transferred to PVDF membranes and blotted with antibodies against interleukin 1 receptor-associated kinase 1 (IRAK1; Invitrogen-38-5600), phospho IRAK1–Thr209, (Invitrogen-PA5-38633), transforming growth factor beta-activated kinase 1 (TAK1; Invitrogen-700 113), phospho TAK1–Thr184/187 (Invitrogen-MA5-15073), TGF-beta-activated kinase 1 (TAB1; Invitrogen-PA5-28683), TNF receptor-associated factor 6 (TRAF6; Invitrogen-PA5-29622), and cyclin D1 (Invitrogen-AHF0082). The antibody against β-actin (Invitrogen-MA191399) was used as a loading control.

### Coimmunoprecipitation

Cells were cultured at a density of 100 × 10^4^ cells in a 60-mm culture plate (Tarsons-960020). Four hours before the addition of the TLR3 ligand, the MyD88 inhibitor ST2825 was added and incubated for 24 h. Cells were lysed with non-denaturing lysis buffer (20 mM Tris-HCl, 137 mM NaCl, 1% Triton X-100, 2 mM EDTA) with a protease inhibitor cocktail. The lysate was incubated on ice for 30 min and centrifuged at 10,000 rpm for 20 min at 4°C. The supernatant was incubated with 1 μg of an indicated antibody and Dynabeads (Invitrogen-10003D) overnight at 4°C. The Dynabeads were pelleted down and washed with lysis buffer after overnight incubation. The precipitates were resolved in SDS-PAGE and subjected to Western blotting with the indicated antibodies.

### Statistical Analysis

Statistical analysis was performed with GraphPad Prism version 7. The difference between two groups was determined by two-tailed Student's *t*-test. Two or more groups were compared with one-way ANOVA. A value of *p* < 0.05 was considered as statistically significant.

## Results

### TLR3 Ligand Induces Cell Viability and Proliferation Which Is Restricted by the MyD88 Inhibitor

**Figure 1:**
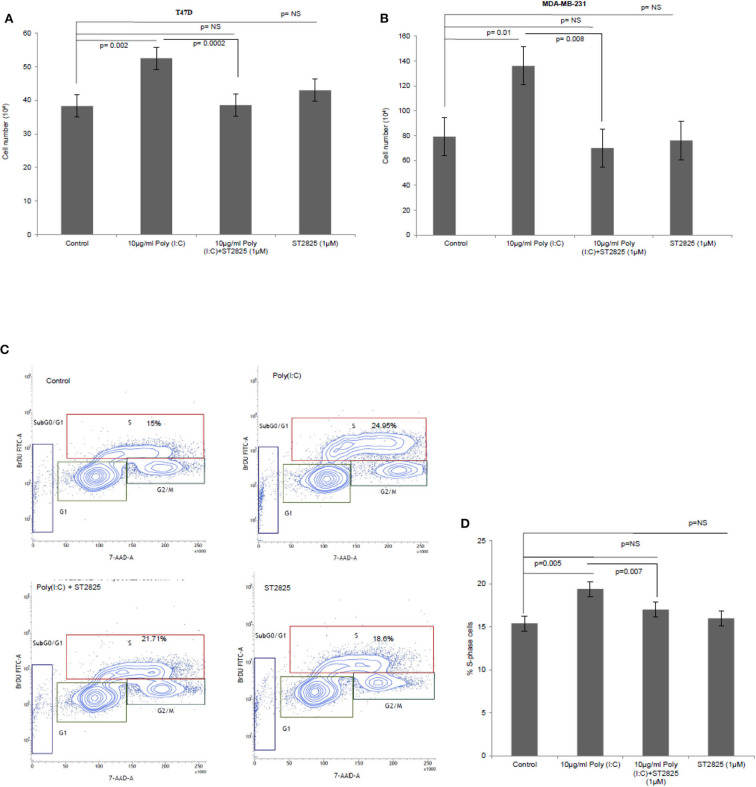
The Toll-like receptor 3 (TLR3) ligand induces cell proliferation and is stunted by myeloid differentiation primary response 88 (MyD88) inhibitor. Breast cancer cells were pretreated with MyD88 inhibitor (1 μM) 4 h prior to the addition of the TLR3 ligand (10 μg/ml) for 24 h before cells were counted. Control indicates cells were not treated with TLR3 ligand or MyD88 inhibitor. Growth kinetic assay. **(A)** T47D and **(B)** MDA-MB-231 showing proliferative effect of TLR3 ligand. Inhibition of MyD88 dimerization restrict the proliferative effect. **(C)** Contour plots for BrdU-cell proliferation assay using T47D cells. Cells were pretreated with MyD88 inhibitor (1 μM) 4 h prior to addition TLR3 ligand (10 μg/ml) for 24 h before labeling with BrdU and detecting by flow cytometry. Contour plots of DNA-7AAD-A vs. log BrdU-FITC showing G_0/_G_1_, S, and G_2_/M gates of control cells, cells treated with TLR3 ligand, cells treated with TLR3 ligand and MyD88 inhibitor, and cells treated with only MyD88 inhibitor. **(D)** Bar graph showing percentage of S-phase-gated cells among the different experimental cell groups following BrdU incorporation. The results are presented as mean ± SD, and *p* < 0.05 is treated as significant.

To verify the alternative TLR3 signaling, breast cancer cells were pretreated with MyD88 inhibitor (ST2825) for 4 h followed by stimulation of TLR3 by the addition of the TLR3 ligand. This was followed by incubation of the cells for 24 h. We have observed a significant increase in cell proliferation of MDA-MB-231 and T47D cells. The MyD88 inhibitor impaired the proliferative effect of TLR3 ligand poly(I:C) in both MDA-MB-231 and T47D cells (Figures 1A,B).

Further, to confirm cellular proliferation, BrdU incorporation assay was undertaken, which revealed a higher percentage of BrdU incorporated in the S-phase cells in TLR3 ligand-treated cells compared to that of untreated cells. The proliferative effect of TLR3 ligand treatment was nullified by treatment with the MyD88 inhibitor (Figures 1C,D). There was no cytotoxic or cell-proliferating effect observed in the cells that have been treated with only the MyD88 inhibitor.

### TLR3 Ligands Stimulate the Expression of Surface TLR3

**Figure 2:**
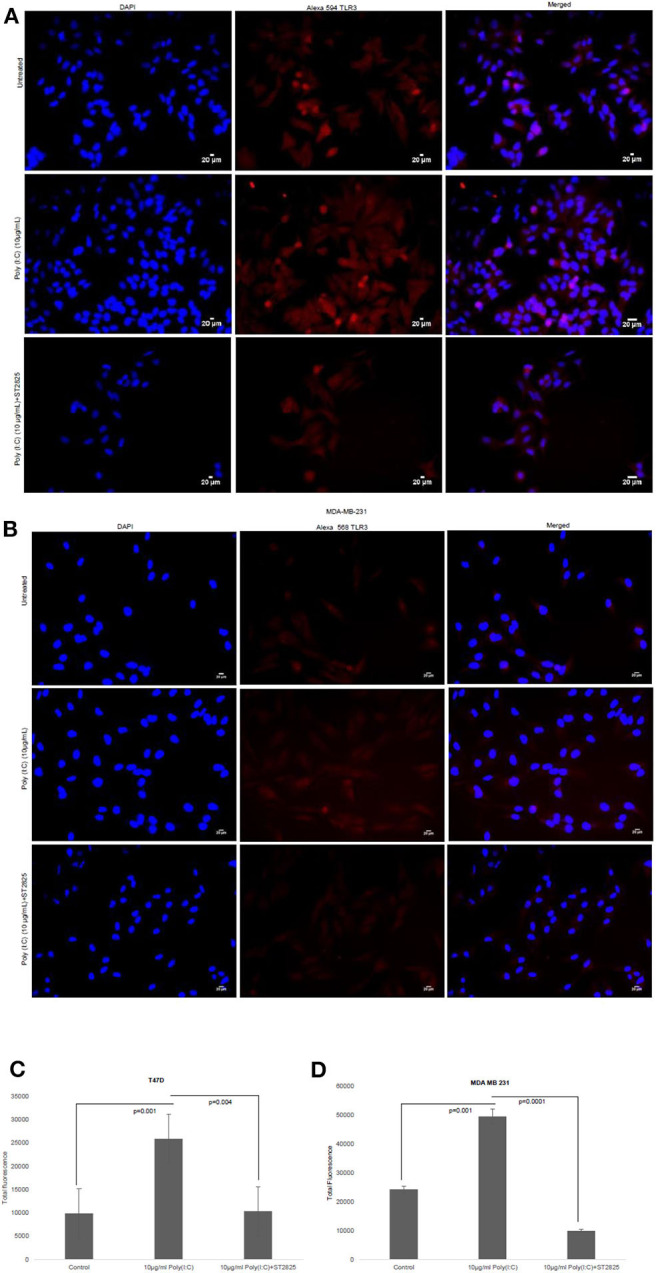
Effect of MyD88 inhibitor on surface localization of TLR3. Fluorescent microscopy image of cells, treated with TLR3 ligand (10 μg/ml) with or without MyD88 inhibitor (1 μM) following immunocytochemical staining with antibody against TLR3 and Alexa 594-tagged secondary antibody and counterstained with DAPI. **(A)** T47D cell and **(B)** MDA-MB-231 cells. **(C,D)** Bar graph showing the localization of TLR3 in cell surface after observation through a microscope and analyses through the ImageJ package for all the experiment groups. The results are presented as mean ± SD (*p* < 0.05 is treated as significant).

To confirm previous findings from our group by Bondhopadhyay et al. (25) about the expression of TLR3 on the cell surface, breast cancer cells were treated with TLR3 ligand poly(I:C). The membrane expression of TLR3 in the absence or presence of exogenous the TLR3 ligand and MyD88 inhibitor was investigated by immunocytochemistry. TLR3 expression was markedly increased in the presence of exogenous TLR3 ligand in comparison to that of the unstimulated cells, while the addition of MyD88 did not have an effect on TLR3 expression (Figure 2).

### MyD88 Inhibitor Reduces the Production of Proinflammatory Cytokine IL-6 in TLR3 Ligand-Treated Breast Cancer Cells

**Figure 3:**
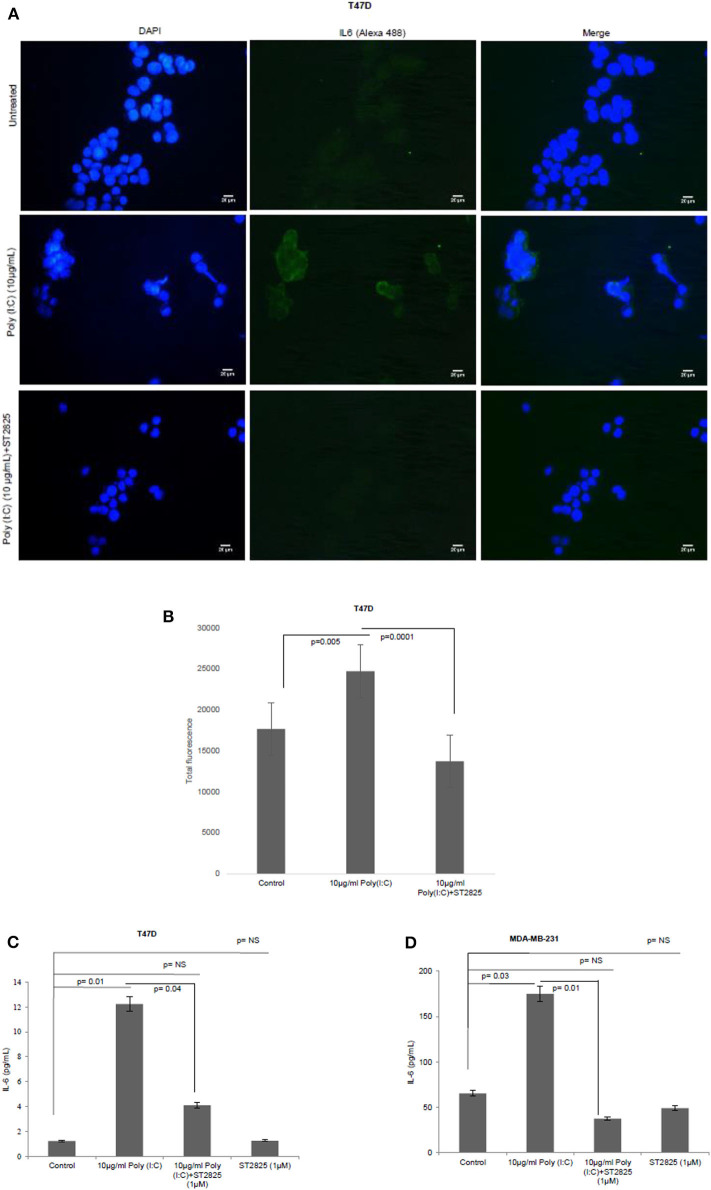
Expression of IL-6 following MyD88 inhibitor and TLR3 ligand treatment. **(A)** Fluorescent microscopy image of T47D cells, treated with TLR3 ligand (10 μg/ml) with or without MyD88 inhibitor (1 μM) following immunocytochemical staining with antibody against IL-6 and Alexa 488-tagged secondary antibody and counterstained with DAPI. Untreated indicates the cells are not treated with TLR3 ligand (magnification, 40X). **(B)** Bar graph showing the expression of IL-6 following observation through a microscope and analyses through the ImageJ software for all the experiment groups. **(C,D)** Expression of IL-6 in the cell culture supernatant as measured through ELISA. The results are presented as mean ± SD (*p* < 0.05 is treated as significant).

To assess whether the TLR3 ligand is able to induce IL-6 production and be reversed, cells were treated with MyD88 inhibitor for 4 h. Accordingly, the MyD88 inhibitor-pretreated cells were challenged with the TLR3 ligand, and the level of IL-6 was determined by immunocytochemistry in the cytoplasm and by ELISA in the condition media. We have observed that TLR3 ligand treatment significantly induces the immunofluorescence and secretion of IL-6 compared to the control group. Pretreatment of MyD88 inhibitor reduced the production of IL-6 in spite of stimulation with the TLR3 ligand (Figure 3).

### MyD88 Inhibitor Attenuates TLR3 Ligand-Induced NF-κB Nuclear Localization

**Figure 4:**
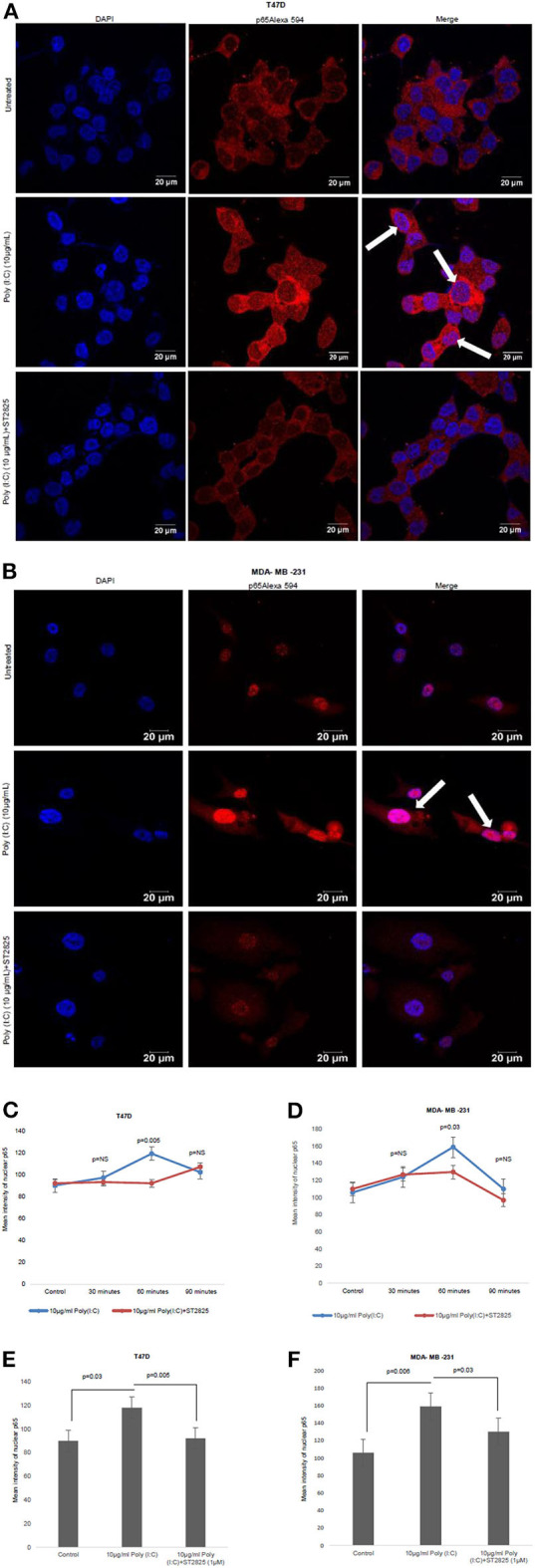
Confocal microscopy for nuclear translocation of p65. **(A)** T47D cells **(B)**, MDA-MB-231 cells were pretreated with MyD88 inhibitor for 4 h prior to addition of TLR3 ligand (10 μg/ml) for 60 min. Cells were stained with antibody against p65 subunit of NF-κB and Alexa 594-tagged secondary antibody and counterstained with DAPI, and image acquired through confocal microscope (magnification, 63X). **(C,D)** Bar graph is presented as mean ± S.D for the quantitative measurements of nuclear localization of NF-κB at 30, 60, and 90 min of stimulation, analyzed through Image J package (*p* < 0.05 is treated as significant). **(E,F)** Bar graph at 60 min of stimulation showing the highest nuclear localization of NF-κB.

In the previous section, we have showed that there was a reduction in the IL-6 expression after the addition of MyD88 inhibitor despite the presence of the TLR3 ligand. Previously, it was reported that early phase activation (0.5–2 h) of NF-κB leads to the production of proinflammatory cytokines (15). In the present work, we had assessed the early phase nuclear localization of p65 subunit of NF-κB. Accordingly, it has been observed that MyD88 inhibitor nullifies TLR3 ligand-induced nuclear localization of p65 (Figure 4). The TLR3 ligand elicits the highest translocation of p65 into the nucleus at a 60-min time point in both the cell lines compared to control untreated cells (Supplementary Figure 1).

### TLR3 Ligand Induced the Expression of Cyclin D1 and Halted by the MyD88 Inhibitor

**Figure 5:**
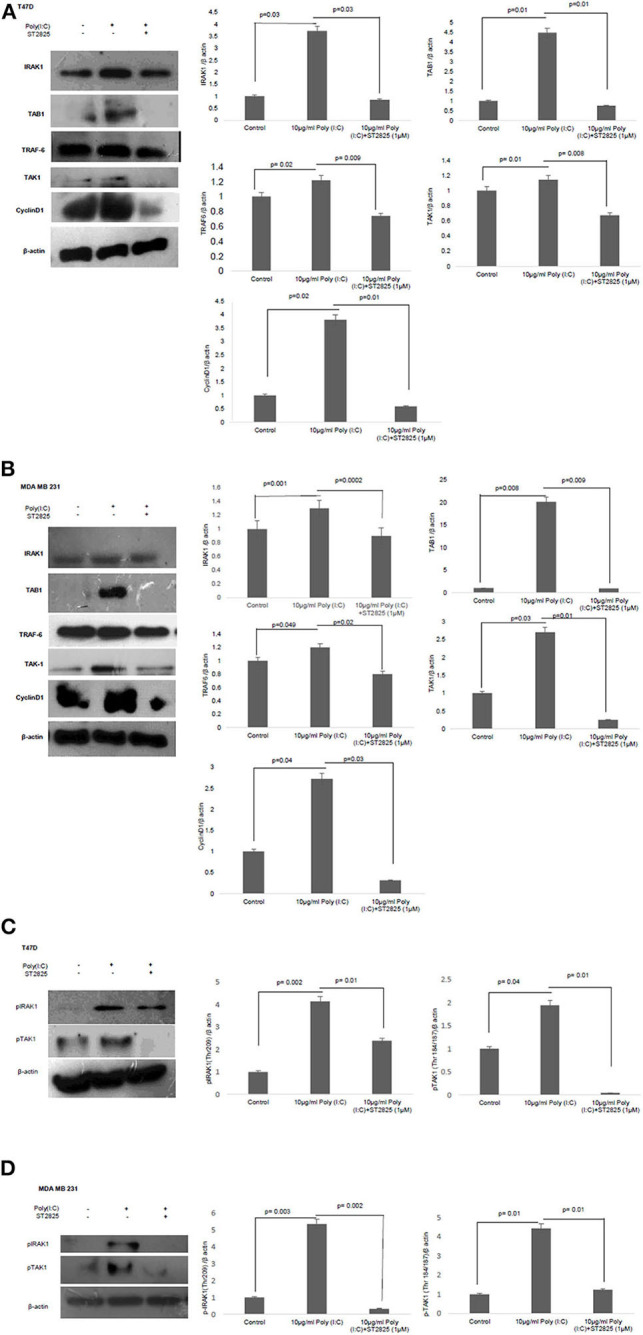
Western blotting for the expression of signaling protein. **(A,B)** Cell lysate were collected and subjected to Western blot assay to estimate the level of expression of interleukin 1 receptor-associated kinase 1 (IRAK1), transforming growth factor beta-activated kinase 1 (TAK1), TGF-beta-activated kinase 1 (TAB1), TNF receptor-associated factor 6 (TRAF6), and cyclin D1. **(C,D)** Expression of pIRAK1 and pTAK1. β-actin was used as loading control. The respective bar graphs are presented as densitometry analysis as mean ± SD of experiments (*p* < 0.05 is treated as significant).

To address that TLR3 mediated cell proliferation, whether regulated through the cyclin D1 gene expression, we investigated the expression of cytosolic cyclin D1 through Western blotting using cell lysate. TLR3 ligand stimulation elevates the expression of cyclin D1. However, the addition of MyD88 inhibitor recorded a decrement in the level of cyclin D1 suggesting a break in the signaling cascade of the TLR3 ligand (Figures 5A,B).

### MyD88 Inhibitor Reduced the Exogenous TLR3 Ligand-Induced Expression of Adaptor Proteins—IRAK1, TAK1, TAB1, and TRAF6

To understand the involvement of an adapter complex to transmit the effect of TLR3 ligand for the expression of cyclin D1, we checked the expression IRAK1, TRAF6, TAK1, TAB1 in the presence or absence of the MyD88 inhibitor. To confirm our hypothesis, the protein level of all the above adaptor proteins was estimated by Western blotting. The significant increase in the expression of IRAK1, TRAF6, TAK1, and TAB1 following the induction of the TLR3 ligand has been observed compared to untreated cells. However, the addition of MyD88 inhibitor ST2825 reduced the level of IRAK1, TRAF6, TAK1, and TAB1 (Figures 5A,B).

### MyD88 Inhibitor Reduces TLR3 Ligand-Mediated Phosphorylation of Adaptor Protein IRAK1 and TAK1

IRAK1, a serine–threonine kinase, was reported to be phosphorylated upon lipopolysaccharide (LPS)-mediated signaling stimulation (27). We have assessed IRAK-1 and TAK1 phosphorylation in MDA-MB-231 and T47D cells in the presence and absence of MyD88 inhibitor following the induction by the TLR3 ligand. Increased level of phosphorylated IRAK1 and TAK1 in the response of exogenous TLR3 ligand addition potentially explains the activation of IRAK1 and TAK1. We found that the MyD88 inhibitor suppressed the TLR3 ligand-mediated level of phosphorylated IRAK1 and TAK1 (Figures 5C,D).

### TLR3 Ligand Induces IRAK1/TRAF6, pIRAK1/TAK1, and TRAF6/TAK1/TAB1 Interactions Which Are Disrupted by MyD88 Inhibitor

**Figure 6:**
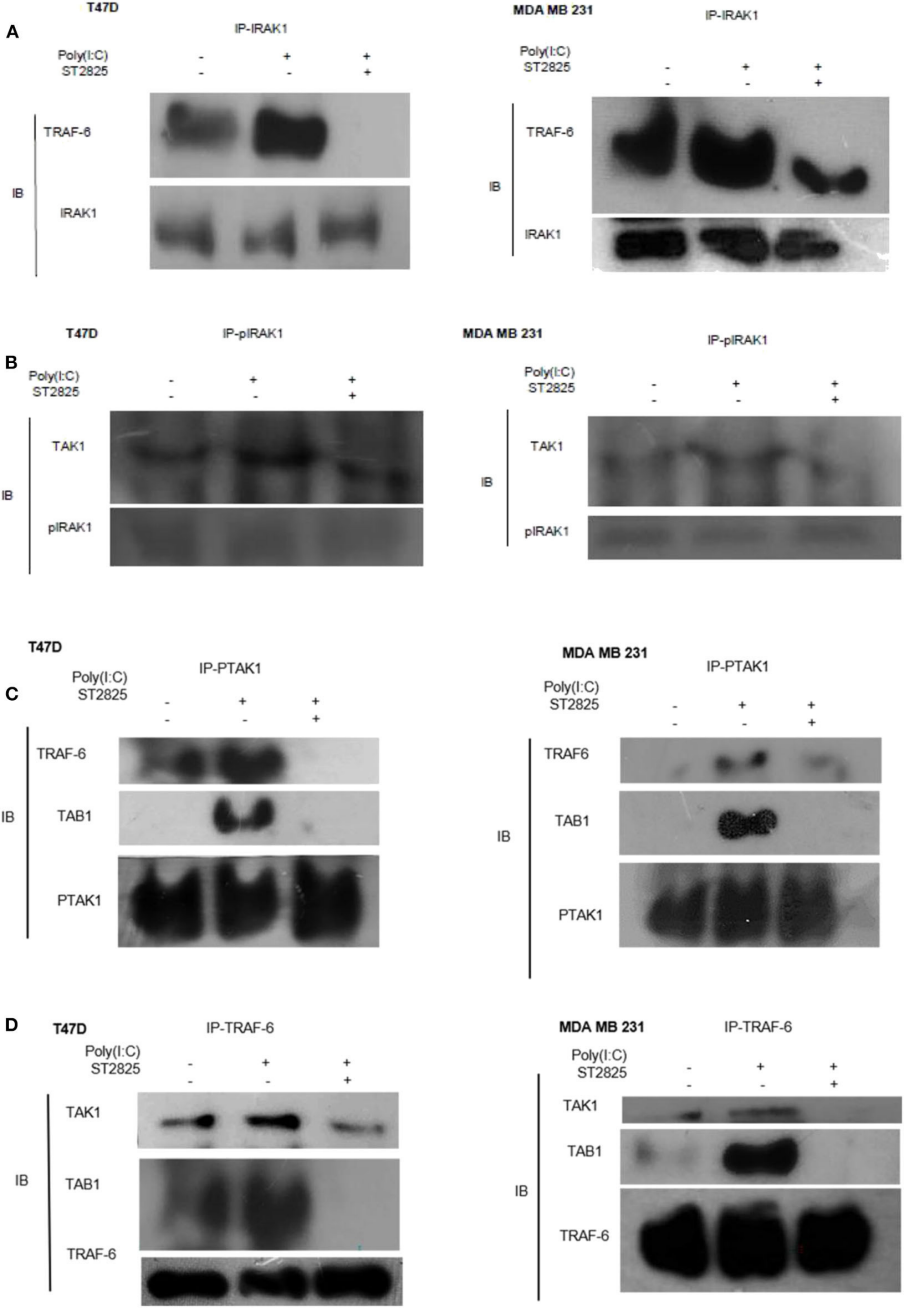
Immunoprecipitation showing the involvement of the signaling complex. **(A)** Signaling complex of IRAK1/TRAF6 was immunoprecipitated with antibodies against IRAK1 followed by Western blotting with anti-TRAF6 and anti-IRAK1 antibodies. **(B)** Signaling complex of pIRAK1/TAK1 was immunoprecipitated with antibodies against pIRAK1 followed by Western blotting with anti-TAK1 and anti-pIRAK1 antibodies. **(C)** Signaling complex TAB1–TRAF6–TAK1 was immunoprecipitated with antibodies against pTAK1 followed by Western blotting using anti-TRAF6, TAB1, and pTAK1 antibodies. **(D)** Signaling complex TAB1–TRAF6–TAK1 was immunoprecipitated with antibodies against TRAF6 followed by Western blotting analysis using anti-TAK1, TAB1, and TRAF6 antibodies.

As there were changes in expression and phosphorylation, further, we addressed the involvement of signaling complex formation of the above adopter proteins. Accordingly, cells were treated with the TLR3 ligand in the presence or absence of the MyD88 inhibitor; thereafter, immunoprecipitation with IRAK1 antibody and immunoblotting with TRAF6 antibody were investigated. In TLR3 ligand-stimulated cells, there was a marked increase in association. In cells pretreated with MyD88 inhibitor before TLR3 ligand addition, the interaction of TRAF6 and IRAK1 was decreased markedly. This suggests that MyD88 inhibition interferes with the formation of the TLR3 ligand-induced IRAK1/TRAF6 complex (Figure 6A).

On the other hand, cell lysate was immunoprecipitated with pIRAK1 antibody and immunoblotted with TAK1 antibody. In TLR3 ligand-stimulated cells, there was a marked increased association. In cells pretreated with MyD88 inhibitor, the interaction of TAK1 with Phospho-IRAK1 was decreased markedly. This suggests that MyD88 inhibitor ST2825 interferes with the association of TLR3 ligand-induced complex of pIRAK1/TAK1 (Figure 6B). It is also worthy to mention that we did not find any immunoprecipitation of TAK1 when precipitated through a non-phosphorylated IRAK1 antibody. As we mention in Figures 5A,B, inhibition of MyD88 dimerization blocks the proinflammatory signaling and lowers the level of TRAF6, TAK1, and TAB1. Herein, we hypothesize that the MyD88 inhibitor interferes with the formation of TLR3 ligand-induced MyD88-mediated TRAF6/TAK1/TAB1 signaling complexes. To evaluate this hypothesis, cells were pretreated with MyD88 inhibitor ST2825 for 4 h prior to stimulation by the TLR3 ligand. Cell lysates were collected and immunoprecipitated with TRAF6 and phosphoTAK1 antibodies, followed by immunoblotting using TAB1, TRAF6, and TAK1 antibodies. In TLR3 ligand-stimulated cells, there was a distinct increase in the association, whereas in the cells pretreated with MyD88 inhibitor, the interaction of TAK1 with either TRAF6 or TAB1 was decreased markedly. This suggests that MyD88 inhibitor (ST2825) interferes with the formation of a signaling complex as mentioned above (Figures 6C,D).

## Discussion

In this present study, we had reported the mechanistic pathway of TLR3 ligand-induced breast cancer cell proliferation through MyD88-mediated gateway. ST2825 is well-established as an MyD88 inhibitor in several studies (2, 28–30) and, hence, was used to address the ligand-mediated alternative cell proliferative action of TLR3. Care was taken that ST2825 is used at a concentration that is neither cytotoxic nor cell proliferative to the breast cancer cell lines (MDA MB 231 and T47D). TLR3 was reported to be expressed only by immune cells, and unstimulated TLR3 mainly resides in the ER. Stimulation of TLR3 with the ligand poly(I:C) causes TLR3 to be translocated from the ER into the endosomal compartment (31). Though regulation of this translocation is reported to be controlled by the UNC93B1 protein, its inhibition has only a partial effect on TLR3-mediated signaling (1). Further, cell surface expression of TLR3 has been reported in a variety of cells such as pulmonary cells, hepatocytes, breast cancer, prostate cancer, and epithelial adenocarcinoma (4–6) indicating that its signaling occurs through the plasma membrane. Dynasore, a dynamin inhibitor that inhibits endocytosis of the receptors, only partially affects the poly(I:C)-mediated TLR3 signaling. This suggests that TLR3 signaling may occur independent of ligand internalization (1). Our result shows an increase in surface expression of TLR3 in breast MDA-MB-231 and T47D cells upon TLR3 ligand activation. Addition of MyD88 inhibitor does not have any effect on the level of surface TLR3 expression suggesting that TLR3 signaling occurs from the cell surface in an MyD88-dependent manner.

We have shown in our initial experiments that exogenous stimulation of TLR3 by its ligand promotes cellular proliferation in breast cancer cells (25). However, this proliferative effect has been perturbed by the addition of the MyD88 inhibitor suggesting that the said effect of TLR3 is mediated by MyD88. In the present investigation, TLR3 activation through the TLR3 ligand stimulated the expression of downstream signaling factors, including IRAK1, TRAF6, TAB1, and TAK1, and suppressed the MyD88 inhibitor that correlates with other signaling cascades (32). It has been reported that activation of other TLRs, in contrast to TLR3, can induce the canonical pathway through MyD88-mediated activation of interleukin 1 receptor-associated kinase 1 (IRAK1). Activation of this signaling pathway further regulates IRAK1-mediated activation of TNF receptor-associated factor 6 (TRAF6) and transforming growth factor beta-activated kinase 1 (TAK1) that further causes activation of TGF-beta-activated kinase 1 (TAB1). Thus, our findings are well-correlated with earlier reports on different convergent signaling pathways (3–5, 20–22, 27, 32–40).

Though MyD88 does not have any catalytic activity, its activation causes dimerization leading to the activation of downstream kinases (10). It has been shown that the progression of signaling pathways occur due to the phosphorylation of two key adaptor proteins IRAK1 and TAK1. IRAK1, a serine–threonine kinase, was reported to be phosphorylated via MyD88 upon lipopolysaccharide (LPS) stimulation (27) that also triggers its dissociation from the membrane and translocation into the cytosol. IRAK1 activation is also required for phosphorylation of TAK1 (27). We had observed that activation of IRAK1 leads to its complex formation with TRAF6 and TAK1. Phosphorylation of IRAK1 helps in dissociation of the TRAF6 complex from the membrane and may facilitate formation of TRAF6, TAK1, and TAB1 complex in the cytosol. Later, the phosphorylated IRAK1 may get ubiquitinated and degraded as suggested by other research groups (27, 32–34). Thus, dissociation of the complex from the membrane may lead to phosphorylation of TAK1, as shown by research groups in other signal pathways (33). In our study, the level of phosphorylation of IRAK1 and TAK1, as well as the association of signaling complex IRAK1/TRAF6, pIRAK1/TAK1, and TRAF6/TAK1/TAB1, was found to be elevated upon TLR3 induction. However, the level of phosphorylation as well as the interaction and formation of signaling complexes was found to be reduced by the administration of MyD88 inhibitor. These findings indicate that the TLR3 act in the TLR3–MyD88–IRAK1–TRAF6–TAK1 axis to promote cellular proliferation.

In recent studies, TAK1 has been identified as a key regulator of various immune responses and inflammatory reactions that promote tumorigenesis, fibrosis, and multiple inflammatory disorders. Accordingly, we have observed that induction of TAK1 phosphorylation as a MyD88 activation cascade induces NF-κB activation followed by secretion of IL-6. This observation is supported by an earlier study wherein inhibition of TAK1 phosphorylation inhibits IL-6 production through an NF-κB-dependent manner (14). As NF-κB is a dimer composed of p65 and p50 subunits (41–43), activation of this TAK1/TAB complex activates the NF-κB signaling pathway, which induces nuclear localization of p65 (44). In our study, we had recorded an early phase activation of NF-κB that had triggered IL-6 release by TLR3 ligand induction, but had shown a downregulation following inhibition of MyD88. LPS induction in mice leads to biphasic stimulation of NF-κB. In the early phase activation (0.5–2 h), the production of proinflammatory cytokines, tumor necrosis factor (TNF), and IL-1β is seen, whereas the late phase activation (8–12 h) is associated with expression of cyclooxygenase 2-derived anti-inflammatory prostaglandins and the anti-inflammatory cytokines and transforming growth factor-β1 (15). Our observation is well-correlated with this report wherein an early phase activation leading to production of proinflammatory cytokines is observed herein.

The induction of breast cancer cell lines (MDA-MB-231 and T47D) with the TLR3 ligand induces cellular proliferation through an MyD88-dependent manner via induction of proinflammatory cytokine IL-6 and cyclin D1. The addition of MyD88 inhibitor disrupts the signaling pathway that leads to a decreased level of IL-6 secretion as well as a decrease in cyclin D1 activation. Cyclin D1 controls cell cycle progression through the G_1_ phase and G_1_-to-S transition (36). Induction of IL-6 has been reported to stimulate cyclin D1 promoter (36). Cyclin D1 has been reported to be induced during MyD88–TRAF6 and TAK-1 signaling pathway via the NF-κB–cyclin D1–STAT 3 pathway (37) and cyclin D1 exported from the nucleus to the cytoplasm during the S phase of the cell cycle (38). TLR3 ligand stimulation elevates the expression of cyclin D1. However, in the present study, it has been observed that addition of MyD88 inhibitor breaks the signaling cascade of the TLR3 ligand, and hence, a decrease in the level of cyclin D1 was recorded. Earlier, it has been reported that there was an elevated IL-6 level in dsRNA-treated TLR3-positive mice, but not in TLR3-negative tumors (45).

TLR3 synthetic ligands were used with conventional chemotherapies or radiotherapy in clinical trials for the treatment of cancer patients (17, 19, 46, 47). This reported that the tumor suppressive and apoptotic effect of TLR3 is achieved predominantly by induction of type I IFN and activation of effector cells, when TLR3 is located within the endosomal compartment (1, 5, 17, 48). TLR3 synthetic ligand poly-ICLC with sorafenib significantly reduces tumor growth, both *in vitro* and *in vivo* in hepatocellular carcinoma (18). The mechanism of the antitumorigenic effect of TLR3 is well-established, wherein the TRIF-dependent classical pathway induces apoptosis in cancer cells through the endosomal compartment (49, 50).

However, there are several contradictory reports on the working mechanism and failure in clinical trials. It has also been reported to promote cellular proliferation in the head and neck and multiple myeloma cell lines via c-Myc and NF-κB, respectively, following TLR3 ligand poly(I:C) stimulation (17). In squamous cell carcinomas of the head and neck (HNSCC), triggering the TLR3 signaling pathway along with cisplatin induces production of the proinflammatory cytokines IFN-β, IL-6, and CCL5 to promote cellular survival (39). In a study on metastatic intestinal epithelial cells (IECs), full-length and cleaved form of surface TLR3 has been reported, but activation of endosomal TLR3 by poly(I:C) neither induced IFN-β production nor induced cell apoptosis that implies toward the cell surface signaling of TLR3 (1). We had previously reported the surface localization of TLR3 and its proliferative effect on breast cancer cells (25). In the present study, we have shown the proliferation of two different types of breast cancer cell lines, viz, a triple-negative breast cancer cell MDA-MB-231 and estrogen receptor-positive cells—T47D cells by induction of the TLR3 ligand, which was downregulated by the addition of the MyD88 inhibitor.

**Figure 7:**
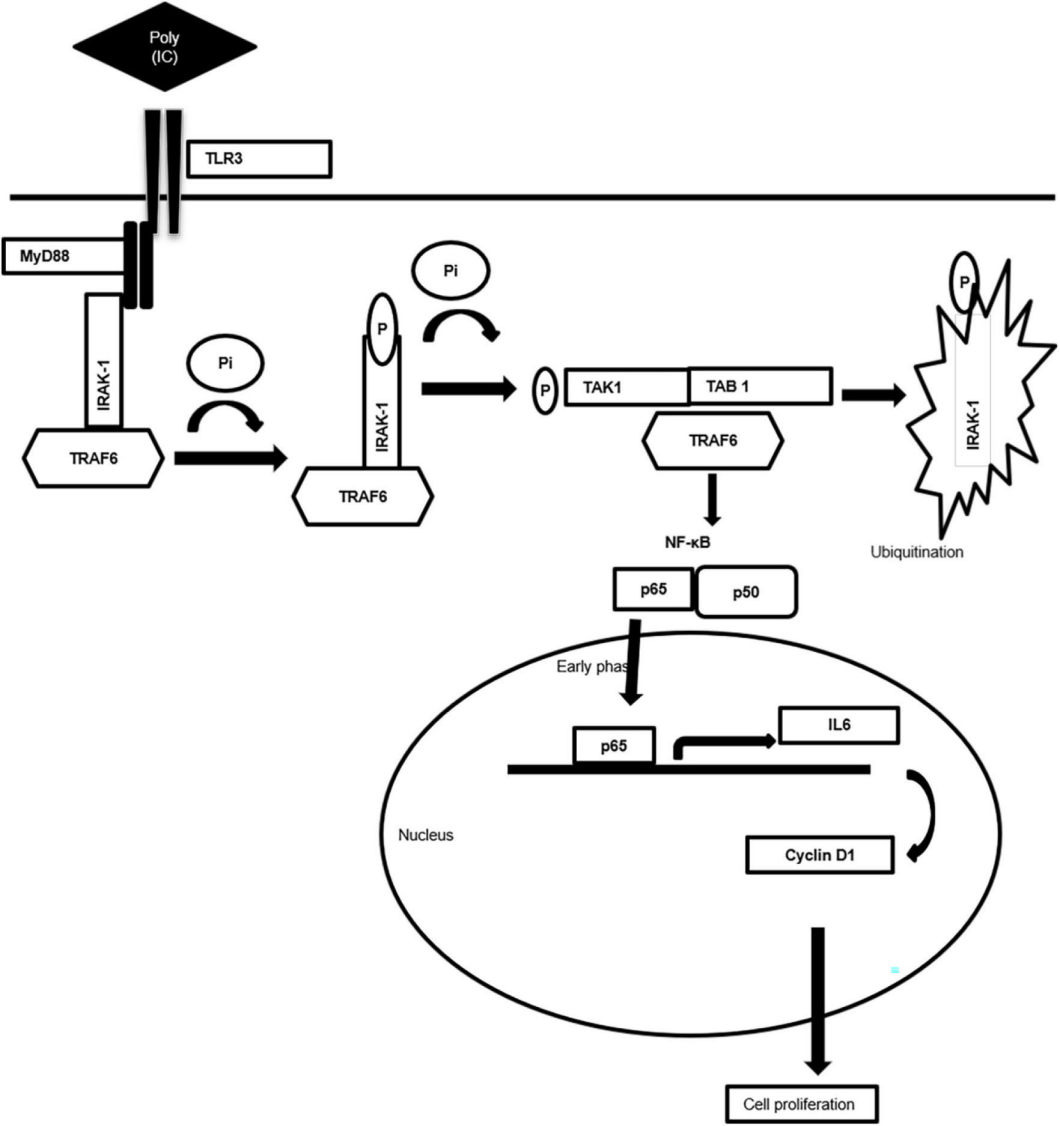
Schematic diagram showing mechanism of MyD88 adopter-mediated surface TLR3 signaling. The diagram illustrating how TLR3 ligand polyinosinic:polycytidylic acid [poly(I:C)] induces the recruitment of the MyD88 complex and activation of downstream signaling cascade. Downstream activation of IRAK-1, TAK1, TRAF6, and TAB1 enables translocation of NF-κB, p65 to the nucleus to induce the secretion of proinflammatory cytokine IL-6 that induces cell proliferation via cyclin D1.

Taken together, the present work generates valuable evidence on the TLR3-mediated alternative signaling of the TLR3–MyD88–IRAK1–TRAF6–TAK1–TAB—NF-κB axis leading to the upregulation of IL-6 and cyclin D1 and culminating in the proliferation of breast cancer cells, a response that is regulated via the MyD88 gateway. Accordingly, a mechanism scheme of alternative TLR3 signal transduction responsible for the proliferation of the cancer cells has been presented (Figure 7). The outcome of the present study will help in better understanding of the differential response observed in therapeutic use of TLR3. Based on the findings, it is recommended that a targeted delivery of the TLR3 ligand to the endosomal compartment, bypassing the MyD88 signaling and subsequently causing activation of the TRIF signaling, can trigger the apoptotic cascade in cancer cells.

## Declaration

The part of the current work has been presented in the ESMO Breast Cancer Conference, Berlin 2019, held on May 2–4, 2019, and appeared in the Abstract book of ESMO Breast Cancer 2019, at the Annals of Oncology, Volume 30. Issue supplement _3.

## Data Availability Statement

The data used to support the findings of this study are available from the corresponding author upon request.

## Author Contributions

AS performed the experiments. RD helped in some experiments. AB designed and supervised the entire study. All authors contributed to the article and approved the submitted version.

## Conflict of Interest

The authors declare that the research was conducted in the absence of any commercial or financial relationships that could be construed as a potential conflict of interest.

## Abbreviations

ANOVA: analysis of variance
dsRNA: double-stranded RNA
ER: endoplasmic reticulum
HMW: high-molecular-weight
MyD88: myeloid differentiation primary response 88
poly(I:C): polyinosinic:polycytidylic acid
TLR 3: Toll-like receptor 3
TRIF: TIR domain-containing adaptor-inducing interferon.
