# Supplementary material for: Myeloid Differentiation Primary Response 88–Cyclin D1 Signaling in Breast Cancer Cells Regulates Toll-Like Receptor 3-Mediated Cell Proliferation
Source: Front Oncol. 2020 Sep 18;10:1780. doi: 10.3389/fonc.2020.01780 (PMC7531238; doi:10.3389/fonc.2020.01780)
Supplement: Supplementary file 1 [file Data_Sheet_1.doc]

**Supplementary File**

**Alternative MyD88 -Cyclin D1 signaling in breast cancer cells regulates TLR3 mediated cell proliferation**

Aradhana Singh^1^, Ranjitsinh Devkar^2#^, Anupam Basu^1^*

From the ^1^Molecular Biology and Human Genetics Laboratory, Department of Zoology, The University of Burdwan, Golapbag Burdwan, 713104, West Bengal, India.

^2^Department of Zoology, Faculty of Science, The M.S. University of Baroda, Vadodara, Gujarat 390002, India

To whom correspondence should be addressed: Prof. Anupam Basu, Dept. of Zoology, The University of Burdwan, Purbo Bardhaman-713104, India [abasu@zoo.buruniv.ac.in](mailto:abasu@zoo.buruniv.ac.in).

#Co-corresponding Author: E-mail: [rv.devkar-zoo@msubaroda.ac.in](mailto:rv.devkar-zoo@msubaroda.ac.in)

**Supplementary Fig 1**:   ST2825 attenuates poly(I:C) induced MyD88 dependent early phase activation of NF-κB activation for nuclear translocation of p65 in time dependent manner. **a** T47D cells and **b** MDA-MB-231 cells were pre-treated with ST2825 for 4 hours prior to addition of poly(10µg/mL) for 30 minutes, 60 minutes and 90 minutes. Cells were stained with antibody against p65 subunit of NF-κB and Alexa 594 tagged secondary antibody and counterstained with DAPI and analyzed acquired through confocal microscope.
